# Licorice-derived prenylated chalcones alleviate knee osteoarthritis via multi-target modulation of PI3K-Akt, MAPK and HIF-1α signaling: an integrative UPLC-Q-TOF-MS/MS, network pharmacology and molecular dynamics study

**DOI:** 10.3389/fbinf.2026.1805739

**Published:** 2026-05-21

**Authors:** Wenqiang Qian, Jiangyi Zeng

**Affiliations:** 1 School of Traditional Chinese Medicine, Jiangxi University of Traditional Chinese Medicine, Nanchang, Jiangxi, China; 2 Department of Smart Minimally Invasive Orthopedics, Nanchang Hongdu Hospital of Traditional Chinese Medicine, Nanchang, Jiangxi, China

**Keywords:** knee osteoarthritis, licorice, molecular dynamics, network pharmacology, prenylated chalcones

## Abstract

Knee osteoarthritis (KOA) remains a leading cause of global disability, yet no disease-modifying osteoarthritis drug (DMOAD) has been approved. This exploratory computational study aimed to elucidate the potential bioactive basis and mechanism of action of a clinically used licorice extract against KOA. UPLC-Q-TOF-MS/MS profiling identified 1,071 metabolites, among which prenylated chalcones—including glabrene, 4′-O-methylglabridin, butein and echinatin—were selected as candidate chemotypes for further investigation based on their known pharmacological activities and relative abundance. Network pharmacology revealed 309 overlapping targets between these constituents and KOA-related genes; topological analysis identified TNF, AKT1, GAPDH and HIF1A as highly connected nodes. Functional enrichment indicated that PI3K-Akt, MAPK and HIF-1α pathways are among the principal predicted intervention axes. Molecular docking showed favorable predicted binding energies (≤−8.4 kcal mol^-1^) of the key chalcones for the hub proteins, and 100-ns GROMACS simulations confirmed structurally stable binding sustained by persistent hydrogen bonds and low residue flexibility (RMSF <4 Å). Collectively, these computational findings suggest that licorice prenylated chalcones may exert multi-target, multi-pathway effects potentially relevant to suppressing inflammation, apoptosis and extracellular-matrix degradation in joint tissues. These findings provide a hypothesis-generating framework for developing prenyl-chalcone-based DMOADs and demonstrate the utility of integrating untargeted metabolomics with network pharmacology for botanical drug discovery hypothesis generation.

## 1 Introduction

Knee osteoarthritis (KOA), the most prevalent form of degenerative joint disease, is characterized by progressive cartilage erosion, sub-chondral bone remodeling, synovial inflammation and peri-articular muscle weakness (Geng et al., 2023). It currently affects approximately 250 million people worldwide, with its incidence rising steadily as populations age and obesity becomes more common (Shi et al., 2025). Beyond pain and stiffness, KOA is a leading cause of mobility limitation, loss of working capacity and decreased quality of life, generating enormous socioeconomic burdens (da Costa et al., 2021). Despite the huge medical need, no registered disease-modifying osteoarthritis drug (DMOAD) is yet available (Chen et al., 2021). Recommended pharmacological options—paracetamol, non-steroidal anti-inflammatory drugs (NSAIDs), intra-articular corticosteroids or hyaluronate—merely palliate symptoms and are frequently accompanied by gastrointestinal, renal or cardiovascular adverse events (Quicke et al., 2022; Wilsher, 2022). Consequently, there is an urgent demand for safe, multi-target and cartilage-protective agents that can intervene in the complex pathogenesis of KOA (Somaiya et al., 2024).

Traditional Chinese Medicine (TCM) has accumulated centuries of clinical experience in managing “Bi-syndrome”, a disorder that overlaps modern definitions of osteoarthritis (Huang et al., 2025; Zheng et al., 2021). Among TCM herbs, *Glycyrrhiza* species (licorice) are among the most widely prescribed, not only as a “harmonizing” adjuvant but also as an active anti-rheumatic remedy (Li et al., 2023; Shang et al., 2022). Licorice contains a broad array of flavonoids, triterpenoid saponins, coumarins and stilbenes; many of them possess anti-oxidant, anti-inflammatory, anti-catabolic and even pro-anabolic activities in chondrocytes and synoviocytes (Li et al., 2023). In particular, prenylated chalcones—exemplified by licochalcone A, echinatin and their hydrogenated derivatives—have been experimentally demonstrated to suppress NF-κB and MAPK signaling (Ahmed et al., 2025; Fan et al., 2025), inhibit matrix metalloproteinase (MMP)-13 and aggrecanase-2 (Yang et al., 2025), and downregulate the NLRP3 inflammasome in IL-1β-stimulated chondrocytes (van Dinteren et al., 2022). Although these fragmentary data imply a potential chondro-protective profile, a holistic understanding of how licorice, and especially its prenylated chalcones, might modulate the multifactorial networks driving KOA remains elusive (Kanehisa et al., 2023).

Network pharmacology, an integrative strategy that aligns chemical profiling, target prediction and pathway enrichment, is increasingly used to decode the systemic actions of botanical drugs and generate testable hypotheses (Singh et al., 2022; Xu et al., 2024). Coupling this approach with untargeted metabolomics allows an unbiased inventory of the constituents present in botanical extracts. Moreover, molecular docking and dynamics simulations provide atomistic insight into ligand–target recognition and the temporal stability of the predicted interactions.

In the present exploratory computational study, we first performed comprehensive phytochemical characterization of a clinically used licorice extract by UPLC-Q-TOF-MS/MS to identify its chemical constituents. Based on literature evidence of their pharmacological activities and their relative abundance in the extract, we prioritized prenylated chalcones as candidate bioactive chemotypes for network pharmacology analysis. Integrating target prediction, GO/KEGG enrichment and protein–protein interaction (PPI) analyses (Zhang et al., 2023), we mapped the “compound–target–KOA” landscape and identified PI3K-Akt, MAPK and HIF-1α pathways as principal predicted intervention axes (Hou et al., 2025). Key prenylated chalcones were subsequently examined by molecular docking and 100-ns molecular dynamics simulations against hub targets such as HIF-1α and GAPDH (Li et al., 2025). Our findings provide computational evidence supporting the multi-component, multi-target mechanism by which licorice prenylated chalcones might exert anti-inflammatory, anti-catabolic and pro-survival effects on joint tissues, offering a theoretical basis for developing licorice-based DMOADs (Oo et al., 2021). It is important to emphasize that this study is entirely computational and serves to generate hypotheses for future experimental validation.

## 2 Results

### 2.1 Identification of active components in licorice using UPLC-Q-TOF-MS/MS

**FIGURE 1:**
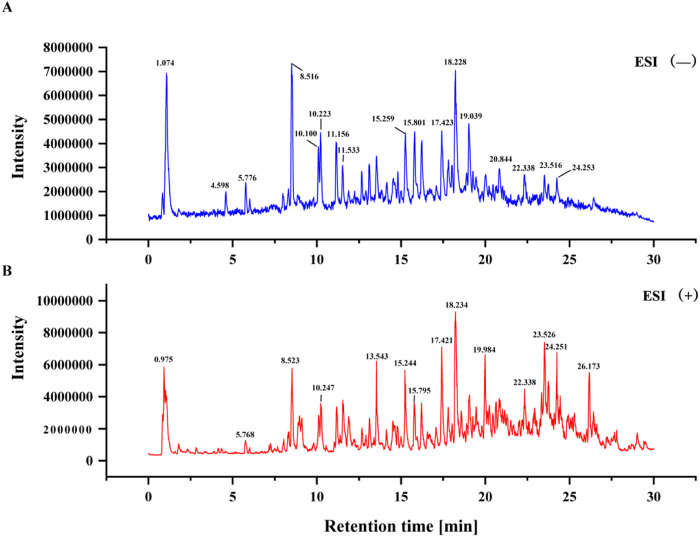
ESI-MS metabolite profiling of licorice extract. **(A)** Total ion chromatogram (TIC) in ESI^−^ mode. **(B)** TIC in ESI^+^ mode.

Licorice samples were analyzed using UPLC-Q-TOF-MS equipped with Analyst TF 1.7.1 and PeakView 1.2 software for data acquisition (Gerona et al., 2018; Colby and Lynch, 2019). Compound identification was performed by matching the acquired mass spectrometry data against the Natural Products HR-MS/MS Spectral Library (version 1.0). Tentative identification was initially conducted based on matching scores assigned to each chromatographic peak, followed by definitive confirmation using both MS and MS/MS spectral data. By integrating multistage fragmentation patterns with high-resolution accurate mass data from natural product databases and relevant literature, a total of 1,071 compounds were identified from the licorice extract. The total ion chromatograms are shown in Figure 1; detailed information, including measured mass, calculated mass, retention time, and peak area for each compound, is provided in Supplementary Table S1. The raw mass spectrometry data (WIFF files) are available for download as a supplementary data file (Data S1).

### 2.2 Classification and characterization of phytochemical constituents

**FIGURE 2:**
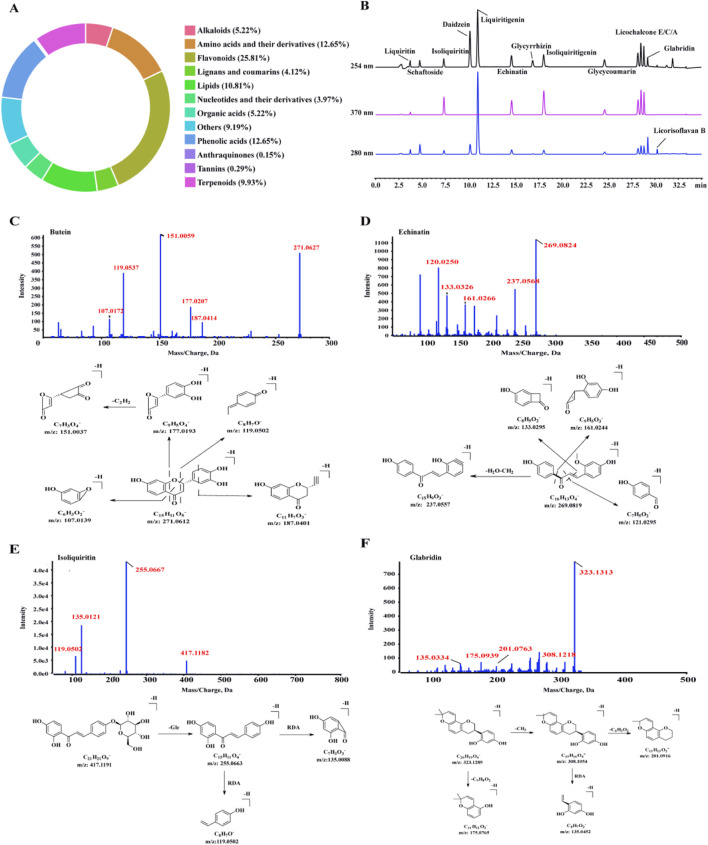
Phytochemical profiling and UHPLC-MS/MS identification. **(A)** Chemical class distribution. **(B)** UHPLC chromatograms of standards at 254, 280, and 370 nm. **(C–F)** Representative MS/MS spectra and fragmentation pathways.

By comparing with reference standards and MS databases, the major chemical classes identified in licorice included flavonoids (25.81%), amino acids and derivatives (12.65%), phenolic acids (12.65%), terpenoids (9.93%), and lipids (10.81%), among others (Figure 2A). Notably, several pharmacologically active constituents—such as liquiritin, isoliquiritin, echinatin, glycyrrhizin, licochalcone A/C/E, and glabridin—were detected (Figure 2B). Among these, prenylated chalcones (including glabrene, 4′-O-methylglabridin, butein, and echinatin) were prioritized for subsequent network pharmacology analysis based on: (i) their reported anti-inflammatory and chondroprotective activities in existing literature; (ii) their relative abundance indicated by peak areas; and (iii) their established oral bioavailability and pharmacokinetic profiles in previous studies. Further MS/MS analysis elucidated the fragmentation pathways and structural characteristics of representative compounds, including butein, echinatin, isoliquiritin, and glabridin (Figures 2C–F). Detailed structural information for these prenylated chalcones, including SMILES strings, molecular formulas, molecular weights, PubChem compound identifiers (CIDs), and reported bioactivities, is provided in Supplementary Table S2.

### 2.3 Network pharmacology analysis

**FIGURE 3:**
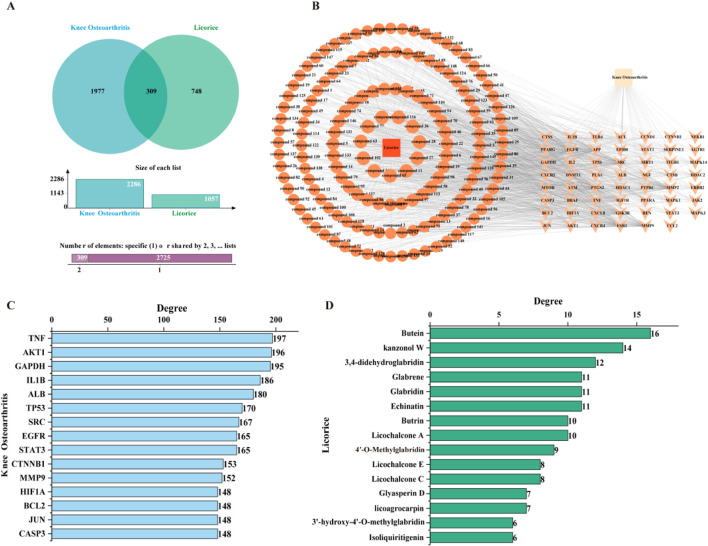
Network pharmacology analysis. **(A)** Venn diagram of overlapping targets (licorice: n = 1,057; KOA: n = 2,286). **(B)** Compound–target–KOA network. **(C)** Top 15 targets by degree centrality. **(D)** Top 15 compounds by degree centrality.

A “compound–target–disease” network was constructed to identify potential anti-KOA bioactive components and their corresponding targets. Venn diagram analysis revealed 309 overlapping targets between licorice-derived prenylated chalcones and KOA-related genes (Figure 3A; Supplementary Table S3). A “compound–target–KOA” interaction network was established to visualize multicomponent, multitarget relationships (Figure 3B).

Critical considerations regarding hub target selection. Topological analysis identified TNF, AKT1, and GAPDH as highly connected nodes (Figure 3C), while key candidate compounds included butein, kanzonol W, and 3,4-didehydroglabridin (Figure 3D). We acknowledge that GAPDH, identified as a highly connected node, is a housekeeping enzyme with pleiotropic functions, and its appearance in network analyses may reflect high topological connectivity rather than specific therapeutic relevance in KOA. These computational predictions require experimental validation to establish biological relevance. The hub targets identified here should be considered as candidates for further investigation rather than validated therapeutic targets.

### 2.4 Functional enrichment and PPI network analysis

**FIGURE 4:**
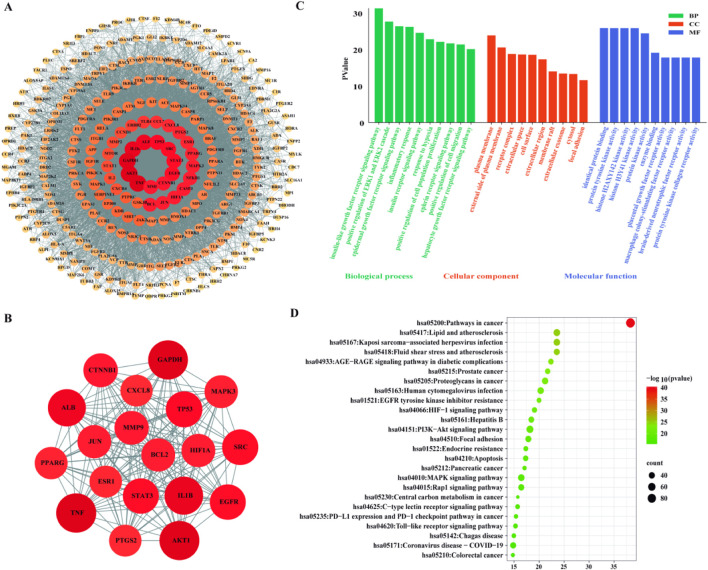
Functional enrichment and PPI network. **(A)** PPI network (192 nodes). **(B)** Core subnetwork of 15 hub genes. **(C)** Top 10 GO terms. **(D)** KEGG pathway enrichment with exact FDR values.

A total of 301 potential target proteins were imported into the STRING database to construct a protein–protein interaction (PPI) network. Using a confidence score threshold of ≥0.900, a refined network of 192 nodes was generated and visualized using Cytoscape 3.10.3 (Figure 4A). Key hub proteins—such as HIF1A, JUN, BCL2, MMP9, CTNNB1, EGFR, STAT3, SRC, TP53, ALB, IL1B, GAPDH, AKT1, and TNF—were identified based on degree centrality (Figure 4B) (Dieden et al., 2024).

GO and KEGG enrichment analyses revealed that these targets were significantly enriched in pathways related to PI3K-Akt signaling (FDR = 1.45E-08), MAPK signaling (FDR = 3.21E-06), HIF-1 signaling (FDR = 8.55E-05), apoptosis, and inflammatory responses (Figures 4C,D). Among these, the PI3K-Akt signaling pathway was the most significantly enriched based on FDR ranking, suggesting its potential central role in the hypothesized therapeutic effects of licorice against KOA.

### 2.5 Molecular docking validation

Binding site definition. Prior to docking, binding sites were defined for each target protein as follows: (i) for proteins with co-crystallized ligands (HIF1A, MMP9), the binding pocket was centered on the native ligand position; (ii) for proteins with literature-documented functional sites (TNF at the TNF dimer interface, AKT1 at the ATP-binding pocket), these sites were used; (iii) for GAPDH, the NAD^+^-binding site was selected based on its functional importance (Ren et al., 2018). Grid boxes (20 Å × 20 Å × 20 Å) were centered on these sites to ensure comprehensive coverage.

**FIGURE 5:**
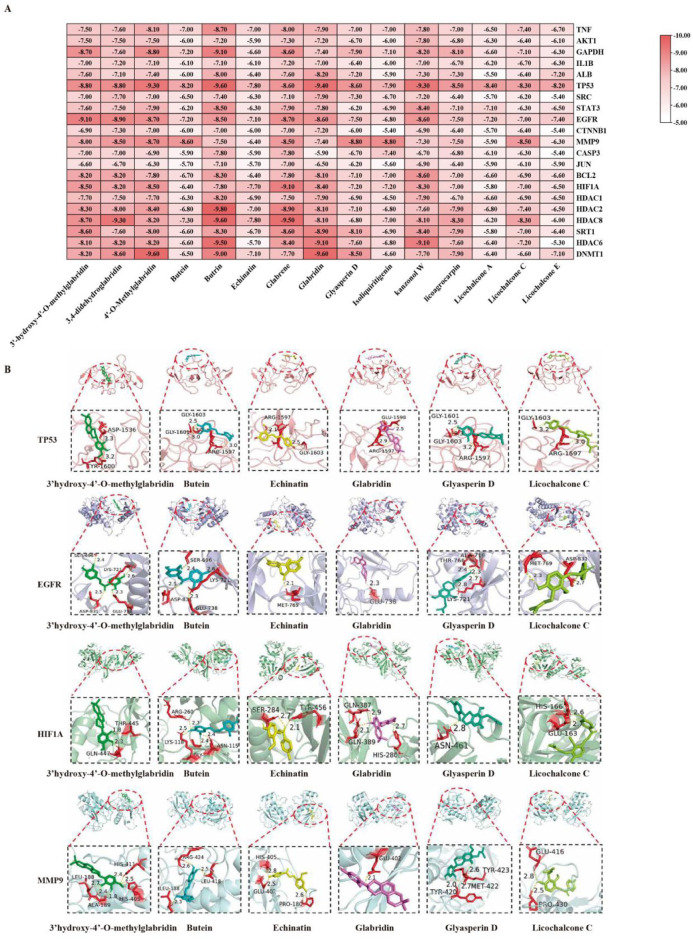
Molecular docking analysis. **(A)** Heatmap of predicted binding energies. **(B)** Docking poses of selected complexes.

Molecular docking was performed between 16 representative compounds and 20 key target proteins. The majority of compounds exhibited favorable predicted binding energies, with docking scores ≤ −6.0 kcal/mol (Figure 5A). Notably, complexes such as 4′-O-methylglabridin–GAPDH and glabrene–HIF1A showed predicted binding energies ≤ −8.4 kcal mol^-1^. The values reported represent predicted binding energies from molecular docking calculations, not experimentally determined binding constants or nanomolar affinities. Representative docking poses showed hydrogen bonding and hydrophobic interactions (Figure 5B).

### 2.6 Molecular dynamics simulations

**FIGURE 6:**
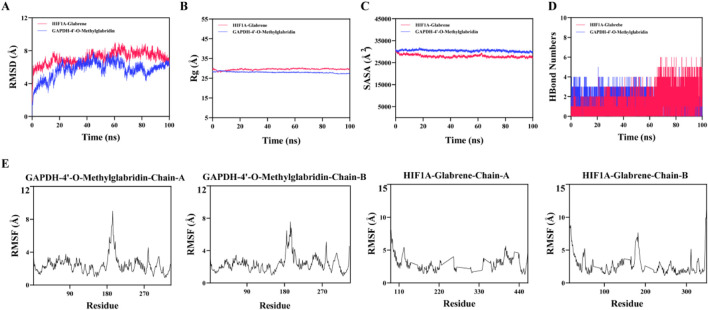
MD simulation analysis. **(A)** RMSD, **(B)** Rg, **(C)** SASA, **(D)** hydrogen bond count, and **(E)** RMSF plots for HIF1A–glabrene and GAPDH–4′-O-methylglabridin complexes.

To assess the structural stability of key compound–target complexes, molecular dynamics (MD) simulations were conducted for HIF1A–Glabrene and GAPDH–4′-O-Methylglabridin. RMSD values stabilized at approximately 6.5 Å and 6.3 Å, respectively, following initial equilibration (Figure 6A). While these RMSD values indicate structural convergence, we acknowledge that absolute values of ∼6.3–6.5 Å suggest substantial backbone fluctuations rather than tight rigid binding, which is typical for protein-ligand complexes of this size and flexibility. Radius of gyration (Rg) and solvent-accessible surface area (SASA) analyses showed consistent compactness and minimal conformational changes after the initial 20-ns equilibration period (Figures 6B,C). Hydrogen bond analysis revealed an average of three persistent bonds, supporting reasonably stable binding interactions (Figure 6D). RMSF values remained <4 Å for the majority of residues, indicating moderate residue flexibility (Figure 6E).

## 3 Discussion

### 3.1 Identification of prenylated chalcones in licorice by UPLC-Q-TOF-MS

UPLC-Q-TOF-MS analysis enabled rapid identification of prenylated chalcones in licorice. The total ion chromatogram exhibited multiple characteristic peaks, and through accurate mass measurement and fragmentation pattern analysis, several typical prenylated chalcones were identified. These results are consistent with previous studies reporting prenylated chalcones in licorice (Montoro et al., 2011; Lu et al., 2025). Glabrene has been shown to possess anti-inflammatory and antioxidant activities (Wahab et al., 2021), while 4′-O-methylglabridin has been reported to have potential protective effects on cartilage. We wish to emphasize that these literature reports provide a rationale for prioritizing these compounds, but do not establish that these specific compounds are responsible for anti-KOA effects in our extract.

### 3.2 Network pharmacology analysis of prenylated chalcones as potential KOA modulators

Network pharmacology analysis revealed that prenylated chalcones from licorice may interact with multiple targets and pathways relevant to KOA pathogenesis. The constructed network showed that glabrene and 4′-O-methylglabridin are predicted to interact with targets involved in inflammation, apoptosis, and extracellular matrix metabolism—core pathological processes of KOA.

Enrichment analysis indicated significant enrichment in TNF, NF-κB, and PI3K-Akt signaling pathways. These computational predictions suggest hypothetical mechanisms by which prenylated chalcones might modulate KOA-related processes, but require experimental validation to establish biological relevance. The PI3K-Akt pathway is involved in cell survival and metabolism; its dysregulation is associated with chondrocyte apoptosis and matrix destruction (Yan et al., 2019). However, we caution that pathway enrichment does not demonstrate actual pathway modulation, and these predictions remain hypothetical until tested experimentally.

### 3.3 Molecular dynamics simulation: implications and limitations

Molecular dynamics simulation was employed to further investigate the predicted binding stability between prenylated chalcones and their candidate targets (Tarrahimofrad et al., 2020). The results suggest that the HIF1A–glabrene and GAPDH–4′-O-methylglabridin complexes maintain reasonable structural stability over the simulation timescale, with persistent hydrogen bond interactions.

Target druggability considerations. We note that GAPDH is a ubiquitous housekeeping enzyme central to glycolysis, and its targeting raises concerns about systemic metabolic effects and off-target toxicity. HIF1A (HIF-1α) is a transcription factor with complex regulatory roles in oxygen homeostasis. The identification of these proteins as highly connected nodes in our network analysis does not establish them as viable therapeutic targets for KOA, and any drug development strategy would need to carefully consider specificity and potential adverse effects.

### 3.4 Limitations and future directions

This study has several important limitations that must be acknowledged:

Absence of experimental validation. All results presented are computational predictions. No cell-based assays, biophysical binding measurements, or animal studies were performed to validate the predicted compound–target interactions, pathway modulations, or therapeutic effects. This study is explicitly positioned as hypothesis-generating research, and all findings require experimental validation before any therapeutic implications can be considered.

Computational limitations. Target prediction algorithms have inherent false positive and false negative rates. Network topology metrics (degree centrality) do not necessarily reflect biological importance or druggability. Docking scores provide approximate rankings of binding likelihood but do not accurately predict binding affinities. The 100-ns MD simulations capture limited timescales and may miss important conformational dynamics.

Bioavailability considerations. This study does not address absorption, distribution, metabolism, excretion, or toxicity (ADMET) properties *in vivo*. While we used predicted ADME properties to prioritize compounds, actual bioavailability and tissue penetration to joint cartilage remain unknown.

Future research directions. To advance these findings, we propose: (i) biophysical validation of key compound–target interactions using surface plasmon resonance or microscale thermophoresis; (ii) cell-based assays in chondrocytes and synoviocytes to test effects on inflammation, matrix degradation, and cell survival; (iii) extended MD simulations with binding free energy calculations (MM/PBSA, MM/GBSA); (iv) pharmacokinetic studies to assess bioavailability and joint tissue penetration; (v) animal model studies to evaluate efficacy and safety.

## 4 Materials and methods

### 4.1 Chemicals and reagents

Licorice (*G. uralensis*, *G. glabra* and *G. inflata*) slices were provided by Hongdu Hospital of Traditional Chinese Medicine (Nanchang, China). Reference standards (purity ≥98%) were obtained from Shanghai Yuanye Bio-Technology Co., Ltd. LC-MS grade acetonitrile and methanol were supplied by Thermo Fisher Scientific. Formic acid was acquired from Sigma-Aldrich. Ultrapure water was prepared using a Milli-Q system.

### 4.2 Preparation of licorice extract

Licorice roots were dried at 45 °C, pulverized, and sieved through a 60-mesh sieve. A 10.0 g aliquot was extracted with 100 mL of 70% methanol (v/v) under ultrasonic assistance (300 W, 40 °C) for 60 min (Hao et al., 2020). The extraction was repeated twice; combined extracts were centrifuged at 8000 *g* for 15 min at 4 °C. The supernatant was concentrated to dryness and reconstituted with 50% methanol to 10 mg/mL, then filtered through 0.22 μm PES filters before analysis.

### 4.3 UPLC-Q-TOF-MS/MS analysis

UPLC analysis was performed on an Agilent 1290 Infinity II system with a ZORBAX Eclipse Plus C18 column (2.1 mm × 150 mm, 1.8 μm). Mobile phase: 0.1% formic acid in water (A) and acetonitrile (B). Gradient: 0–5 min, 5%–15% B; 5–15 min, 15%–30% B; 15–25 min, 30%–60% B; 25–30 min, 60%–95% B; 30–32 min, 95% B; 32–33 min, 95%–5% B; 33–35 min, 5% B. Flow rate: 0.3 mL/min; column temperature: 40 °C; injection volume: 2 μL.

MS detection used an Agilent 6545 Q-TOF with ESI source in both positive and negative modes. Parameters: capillary voltage 4.0 kV (ESI^+^)/3.5 kV (ESI^−^); nozzle voltage 1.0 kV; gas temperature 325 °C; drying gas 8 L/min; nebulizer 40 psi; sheath gas 350 °C, 11 L/min. CID collision energy: 10–40 eV. Mass range: m/z 50–1200. Data were processed using MassHunter Workstation Software (version B.08.00) with the Natural Products HR-MS/MS Spectral Library (version 1.0, mass tolerance ≤5 ppm).

### 4.4 Collection of targets

#### 4.4.1 Licorice prenylated chalcone targets

Structures of identified prenylated chalcones (glabrene, 4′-O-methylglabridin, butein, echinatin) were drawn using ChemBioDraw Ultra 14.0 and converted to SMILES. These were submitted to SwissTargetPrediction (probability ≥0.1, *Homo sapiens*) (Daina et al., 2019), TargetNet (similarity ≥0.85) (Yao et al., 2016), and PharmMapper (fit score ≥0.7). Targets were integrated and duplicates removed.

#### 4.4.2 KOA-related targets

Collected from: (i) DisGeNET (score ≥0.3), OMIM, GeneCards (relevance ≥10); (ii) PubMed literature (2018–2024) with search terms (“knee osteoarthritis” OR “KOA”) AND (“target” OR “gene” OR “protein”) AND (“pathogenesis” OR “treatment”); (iii) DrugBank (Wishart et al., 2018) and TTD for targets of existing KOA therapeutics. Duplicates were eliminated.

#### 4.4.3 Screening of common targets

Intersection of licorice prenylated chalcone targets and KOA-related targets was calculated using Venny 2.1 to identify potential therapeutic targets (Hildebrand et al., 2019).

### 4.5 Network construction and analysis

The “compound-target-KOA” network was constructed using Cytoscape 3.10.3. Topology was analyzed with CytoNCA plugin (version 2.1.6); degree centrality identified core compounds and hub targets.

For PPI network, common targets were uploaded to STRING (version 11.5, confidence ≥0.900, *Homo sapiens*) (Szklarczyk et al., 2021). Data were visualized in Cytoscape; MCODE plugin (degree cutoff = 2, node score cutoff = 0.2, k-core = 2, max depth = 100) identified functional modules. Hub genes were determined by degree, betweenness, and closeness centrality.

### 4.6 Functional enrichment analysis

GO and KEGG enrichment used ClusterProfiler (version 4.6.2) in R (version 4.3.1). Significance: FDR <0.01 and P < 0.05. Top 10 GO terms per category and top 20 KEGG pathways were visualized with ggplot2 (version 3.4.4).

### 4.7 Molecular docking

#### 4.7.1 Protein and ligand preparation

Protein structures from PDB: HIF1A (1H2K), GAPDH (1U8F), AKT1 (3QKK), TNF (2AZ5), MMP9 (1GKC). Preprocessing in AutoDockTools 1.5.7: removal of waters and ligands, addition of polar hydrogens, Gasteiger charges. Ligand structures from PubChem, optimized with Gaussian 09 (B3LYP/6-31G(d)), then processed in AutoDockTools.

#### 4.7.2 Docking simulation

AutoDock Vina 1.2.3 was used with grid boxes (20 Å × 20 Å × 20 Å, 0.375 Å spacing) centered on defined binding sites. Exhaustiveness = 32. Docking scores (kcal/mol) evaluated binding affinity; lower values indicate stronger predicted binding. Top conformations were visualized in PyMOL 2.5 (Schiffrin et al., 2020).

### 4.8 Molecular dynamics simulation

#### 4.8.1 System preparation

Top two complexes (HIF1A-glabrene, GAPDH-4′-O-methylglabridin) were simulated using GROMACS 2023.2. Systems were solvated in TIP3P water (dodecahedral box), neutralized with Na^+^. AMBER ff14SB force field for proteins; GAFF for ligands with RESP charges from Gaussian 09.

#### 4.8.2 Simulation protocol

Energy minimization (steepest descent, max force <1000 kJ/mol·nm); NVT equilibration (300 K, 100 ps, V-rescale); NPT equilibration (1 atm, 100 ps, Parrinello-Rahman); production run (100 ns NPT, 2 fs timestep, coordinates saved every 10 ps).

#### 4.8.3 Trajectory analysis

RMSD, Rg, SASA, RMSF, and hydrogen bond occupancy were calculated using GROMACS tools and visualized with Grace 5.1.25 and PyMOL 2.5.

## 5 Conclusion

This integrative computational analysis suggests that licorice-derived prenylated chalcones may modulate key signaling pathways implicated in KOA, providing hypotheses for future experimental validation. UPLC-Q-TOF-MS/MS metabolite profiling identified 1,071 compounds, with prenylated chalcones representing candidate chemotypes with literature-supported bioactivities. Network pharmacology predicted 309 overlapping targets and highlighted PI3K-Akt, MAPK and HIF-1 signaling as potentially relevant pathways. Molecular docking indicated favorable predicted binding energies for key chalcones against highly connected proteins, and MD simulations suggested reasonably stable predicted binding modes. These findings provide a theoretical framework and testable predictions for subsequent experimental studies, demonstrating the value of coupling untargeted metabolomics with network pharmacology for hypothesis generation in botanical drug discovery. All findings remain computational predictions requiring rigorous experimental validation before any therapeutic implications can be drawn.

## Data Availability

The original contributions presented in the study are included in the article/Supplementary Material, further inquiries can be directed to the corresponding author.

## Supplementary material

SUPPLEMENTARY TABLE S1 Identification parameters of chemical constituents detected by UPLC-Q-TOF-MS/MS.

SUPPLEMENTARY TABLE S2 Identified prenylated chalcones: structures (SMILES), molecular weights, PubChem CIDs, and reported bioactivities.

SUPPLEMENTARY TABLE S3 Overlapping targets between licorice prenylated chalcones and KOA.
